# Forkhead box family transcription factors as versatile regulators for cellular reprogramming to pluripotency

**DOI:** 10.1186/s13619-021-00078-4

**Published:** 2021-07-02

**Authors:** Meijun Fu, Huan Chen, Zepo Cai, Yihang Yang, Ziyu Feng, Mengying Zeng, Lijun Chen, Yue Qin, Baomei Cai, Pinghui Zhu, Chunhua Zhou, Shengyong Yu, Jing Guo, Jing Liu, Shangtao Cao, Duanqing Pei

**Affiliations:** 1 Joint School of Life Science, Guangzhou Institutes of Biomedicine and Health, Chinese Academic and Sciences, Guangzhou Medical School, Guangzhou, 511436 China; 2 CAS Key Laboratory of Regenerative Biology, South China Institutes for Stem Cell Biology and Regenerative Medicine, Guangzhou Institutes of Biomedicine and Science, Chinese Academic and Sciences, Guangzhou, 510530 China; 3 University of Chinese Academy of Science, Beijing, 100049 China; 4 Guangdong Provincial Key Laboratory of Stem Cell and Regenerative Medicine, South China Stem Cell and Regenerative Medicine, Guangzhou Institutes of Biomedicine and Science, Chinese Academic and Sciences, Guangzhou, 510530 China; 5 Center for Cell Lineage and Atlas, Bioland Laboratory (Guangzhou Regenerative Medicine and Health Guangdong Laboratory), Guangzhou, 510005 China

## Abstract

Forkhead box (Fox) transcription factors play important roles in mammalian development and disease. However, their function in mouse somatic cell reprogramming remains unclear. Here, we report that FoxD subfamily and FoxG1 accelerate induced pluripotent stem cells (iPSCs) generation from mouse fibroblasts as early as day4 while FoxA and FoxO subfamily impede this process obviously. More importantly, FoxD3, FoxD4 and FoxG1 can replace Oct4 respectively and generate iPSCs with germline transmission together with Sox2 and Klf4. On the contrary, FoxO6 almost totally blocks reprogramming through inhibiting cell proliferation, suppressing the expression of pluripotent genes and hindering the process of mesenchymal to epithelial transition (MET). Thus, our study uncovers unexpected roles of Fox transcription factors in reprogramming and offers new insights into cell fate transition.

**Supplementary Information:**

The online version contains supplementary material available at 10.1186/s13619-021-00078-4.

## Background

Reprogramming somatic cells to induced pluripotent stem cells (iPSCs) by defined transcription factors is a revolutionary concept for biology and medicine, providing not only cellular sources for disease therapy but also models for investigating roles of cell fate decision (Takahashi et al., 2007; Takahashi and Yamanaka, 2006; Yamanaka, 2020). The transcription factor cocktails Oct4, Sox2, Klf4 and Myc or OSKM also named Yamanaka factors cooperatively function to regulate the expression of genes essential for the pluripotency induction (Buganim et al., 2012; Buganim et al., 2014; Chronis et al., 2017). In the last decade, efforts from laboratories around the world proved that acquisition of pluripotency from fibroblasts underwent several important biological processes, such as mesenchyme-epithelial transition, autophagy, metabolism changes and chromatin remodeling (Cao et al., 2018; Guo et al., 2019; Li et al., 2020; Li et al., 2010; Pei, 2008; Wu et al., 2015). Based on these mechanisms discovery and downstream analysis, numbers of transcription factors or chemicals were uncovered to substitute for Yamanaka factors to activate pluripotency and subsequently generate iPSCs. Pluripotency-associated factors, lineage specifiers and epigenetic regulators were capable of replacing the most important reprogramming factors Oct4(Fritz et al., 2015; Gao et al., 2013; Heng et al., 2010; Mai et al., 2018; Shu et al., 2013; Shu et al., 2015). So far, we and other laboratories developed several non-Yamanaka factors derived reprogramming system with varied efficiency (Buganim et al., 2014; Liu et al., 2015; Wang et al., 2019), suggesting that novel molecular function of transcription factors for cell fate control could be uncovered relied on reprogramming system.

Forkhead box (Fox) transcription factors are evolutionarily conserved from yeast to human and have great effects on various biological processes during development such as gastrulation and lineage commitment (Golson and Kaestner, 2016). Mutations in many Fox genes lead to embryonic or perinatal lethality or associated diseases (DeGraff et al., 2014; Kittappa et al., 2007; Zhu, 2016). All members of Fox proteins share conserved winged-helix DNA binding domains but possess different features and functions due to divergent binding partners and cofactors (Golson and Kaestner, 2016). For example, FoxA factors act as pioneer factors to remove histone and open chromatin to control cell identity (Li et al., 2012; Yang et al., 2016; Zaret et al., 2016). FoxO factors were shown to mediate cell cycle arrest and glucose metabolism (Haeusler et al., 2010; Kim et al., 2013). In addition, Fox transcription factors were chosen individually or together with other factors to driven reprogramming or transdifferentiation of fibroblasts into different cell types such as neural precursor cells, hepatocytes and muscle cells (Garcia-Prat et al., 2020; Huang et al., 2014; Sanchez et al., 2014; Xu et al., 2015). For instance, several Fox transcription factors including FOXH1, FOXF1, FOXG1, FOXB1 and FOXA2 were reported to enhance human iPSCs generation (Takahashi et al., 2014). FoxP1 regulates pluripotency induction and maintenance through alternative splicing switch (Gabut et al., 2011). We previously reported chromatin accessibility dynamics and found Fox relevant motif enriched significantly during mouse iPSCs induction (Cao et al., 2018; Guo et al., 2019; Li et al., 2017; Wang et al., 2019). However, their functions in mouse iPSCs generation remain unclear. To address this and find novel pluripotency inducors and regulators, we here systematically studied roles of all memebrs of Fox family transcription factors in mouse pluripotency induction and found that all FoxD factors and FoxG1 accelerate iPSCs induction process from mouse fibroblasts by OSKM while FoxA and FoxO factors impede this process obviously. Moreover, FoxD3, FoxD4 and FoxG1 were able to replace Oct4 respectively and generate iPSCs with germline transmission together with Sox2 and Klf4. Together, this study indicates the importance of Fox family transcription factors in somatic cell reprogramming and increases our knowledge of the interaction of lineage specifiers with pluripotency-associated factors.

## Results

### FoxD3, FoxD4 and FoxG1 replace Oct4 and enhance iPSCs generation from mouse fibroblasts with Sox2 and Klf4

**Fig. 1:**
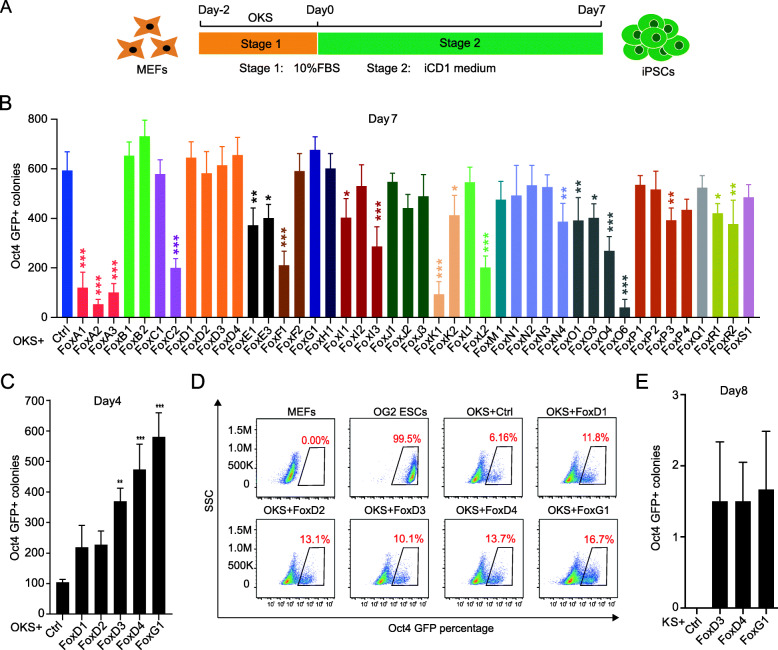
FoxD3, FoxD4 and FoxG1 replace Oct4 respectively and induce miPSCs with KS. **a** Schematics for the process of reprogramming. MEFs were infected with OKS, and then cultured in iCD1 medium. **b** The effects of Fox transcription factors in OKS mediated reprogramming system. The number of GFP positive iPSC colonies was counted at day 7 after induction from three independent biological assays. *, *p* < 0.05; **, *p* < 0.01; ***, *p* < 0.001. **c** Oct4-GFP positive colonies were scored at day 4 post infection; OG-MEFs were transduced with OKS and Fox genes; *n* = 3. **, *p* < 0.01; ***, *p* < 0.001. **d** The proportions of Oct4-GFP positive colonies reflected reprogramming efficiency at day 4 after induction according to flow cytometry analysis. **e** The number of GFP positive iPSC colonies was counted at day 8 after infection with KS and Fox genes; n = 3

In mouse, there are 44 members of Fox transcription factor family: FoxA1, FoxA2, FoxA3, FoxB1, FoxB2, FoxC1, FoxC2, FoxD1, FoxD2, FoxD3, FoxD4, FoxE1, FoxE3, FoxF1, FoxF2, FoxG1, FoxH1, FoxI1, FoxI2, FoxI3, FoxJ1, FoxJ2, FoxJ3, FoxK1, FoxK2, FoxL1, FoxL2, FoxM1, FoxN1, FoxN2, FoxN3, FoxN4, FoxO1, FoxO3, FoxO4, FoxO6, FoxP1, FoxP2, FoxP3, FoxP4, FoxQ1, FoxR1, FoxR2 and FoxS1. To gain insight of any role Fox genes may play in pluripotency, we analyzed the expression of members of Fox transcription factor family in mouse embryonic fibroblast (MEF),mouse embryonic stem cells (mESCs) and during somatic cell reprogramming and mouse early embryo development from E3.5 to 7.5. the results revealed that they varied broadly, indicating these genes might play different roles in pluripotency induction (Supplementary Figure 1A-B). To test this hypothesis, we clone all members of Fox transcription factor family and individually transduced them into OG2-MEFs carrying an Oct4-GFP reporter together with Yamanaka factors Oct4, Klf4 and Sox2 reprogrammed in iCD1 medium (Fig. 1a). After 7 days, we calculated number of Oct4-GFP positive colonies and collected samples for flow cytometer analysis (Fig. 1b and Supplementary Figure 2). The results showed that FoxB subgroups, FoxD subgroups and FoxG1 could facilitate reprogramming while FoxA subgroups, FoxC2, FoxF1, FoxK1 and FoxO6 significantly inhibit somatic cell reprogramming. In addition, we observed cell morphological changes during reprogramming and found that FoxG1 and FoxD subgroup could obviously increase numbers of Oct4-GFP positive colonies at day 4, which was further confirmed by flow cytometer analysis results (Fig. 1c-d). We found that FoxD3/ FoxD4/ FoxG1 promoted cellular proliferation and endo-Oct4 expression obviously at early repgrogramming stage, indicating that they might positively regulate pluripotency (Supplementary Figure 1C, D, E). So, given obvious effect of FoxG1 and FoxD subgroup on reprogramming, we wondered whether FoxG1 and FoxD subgroup could substitude any Yamanaka factor especially for Oct4 which was demonstrated to be most important for iPSCs generation. To test this, we transduced FoxG1 and FoxD subgroup into MEFs with Klf4 and Sox2 in the absence of Oct4. Eventually, several compact and domed Oct4-GFP positive colonies appear at day8 for KS FoxG1, KS FoxD3 and KS FoxD4 but not for KS FoxD1 and KS FoxD2, suggesting four members of FoxD subgroup might have distinct function for pluripotency (Fig. 1e). Taken together, we demonstrated that members of Fox transcription factor family play different roles in mouse somatic cell reprogramming and among which FoxD3, FoxD4 and FoxG1 replace Oct4 and enhance iPSCs generation.

### KS FoxD3/FoxD4/FoxG1 reprogrammed iPSCs are fully pluripotent

**Fig. 2:**
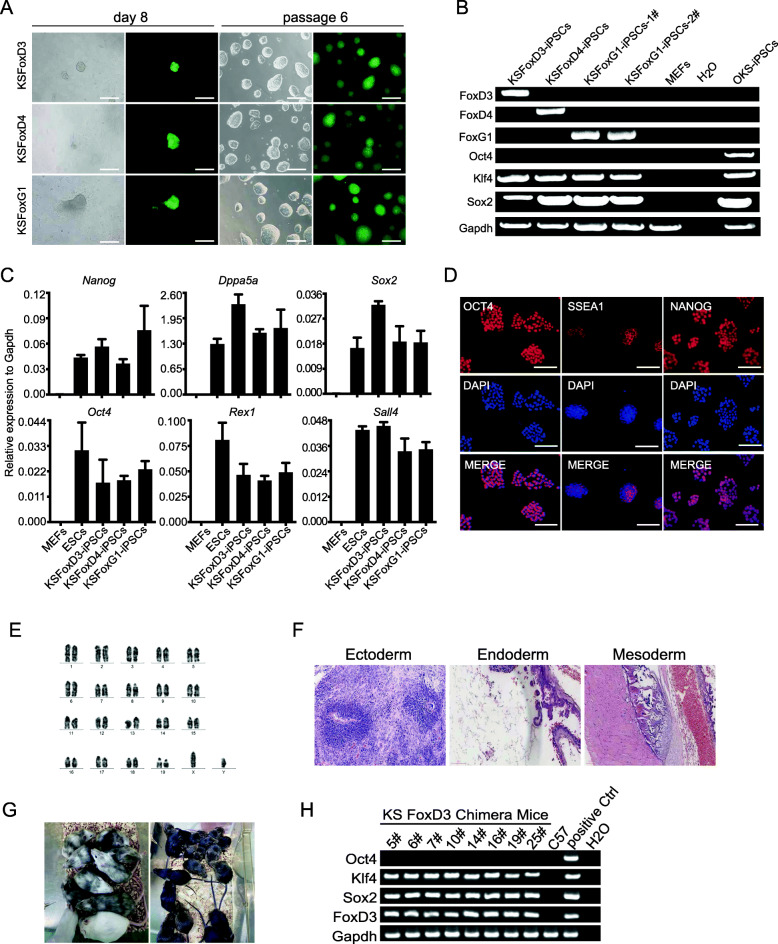
KS FoxD3/ FoxD4/ FoxG1 derived iPSCs resemble mouse ESCs. **a** Representative images of KSFoxs-iPSCs at day 8 after induction. These iPSCs could be passed normally. Scale bar, 250 μm. **b** Genome PCR showed that these KSFox-iPSCs were free of Oct4 transgene contamination. **c** RT-PCR analysis of the expression levels of pluripotent marker genes in KSFoxD3/ FoxD4/ FoxG1 derived iPSCs; n = 3. **d** KSFoxD3 iPSCs colonies expressed pluripotency markers Oct4, Nanog and SSEA-1. DAPI stating was used as control. Scale bar, 100 μm. **e** The KSFoxD3-iPSCs colonies had normal karyotype. **f** Three germ layer reprentative tissues presented in teratomas derived from KSFoxD3-iPSCs. **g** Chimeric mice and germline transmission generated from KSFoxD3-iPSCs. **h** Retroviral integrations of KSFoxD3-iPSCs analyzed by PCR with genomic DNA

To test whether KS FoxD3/FoxD4/FoxG1 reprogrammed iPSCs are fully pluripotent, we performed in vitro and vivo experiments to validate it. We picked Oct4-GFP positive colonies and cultured them in mESC medium supplemented with GSK-3β inhibitor CHIR99021 and ERK inhibitor PD0325901. KS FoxD3/FoxD4/FoxG1 reprogrammed iPSCs could be passaged stably and maintained strong Oct4-GFP expression (Fig. 2a). In addition, genome PCR insertion assay revealed that KSFoxD3, KSFoxD4 and KSFoxG1 iPSCs were free of Oct4 transgene integration (Fig. 2b). These iPSCs colonies also express pluripotent markers like Oct4, Nanog, Sox2, Dappa5a, Sall4 and Rex1 detected by realtime-qPCR (Fig. 2c). Then, we chose KSFoxD3 iPSCs as representative for further detailed characterization. Consistently, KSFoxD3 iPSCs colonies were stained positive for OCT4, SSEA1 and NANOG (Fig. 2d). The KSFoxD3 iPSCs possessed normal karyotype during passages and developed into teratoma with three germ layer tissues when subcutaneously transplanted into NOD SCID mice for 1 month (Fig. 2e-f). We also injected KSFoxD3 iPSCs into blastocysts from ICR mice and obtained chimeric mice with germline transmission (Fig. 2g). Together, our results demonstrated that KSFoxD3, KSFoxD4 and KSFoxG1 iPSCs colonies resemble mouse ESCs and are fully pluripotent.

### FoxO6 inhibits somatic cell reprogramming

**Fig. 3:**
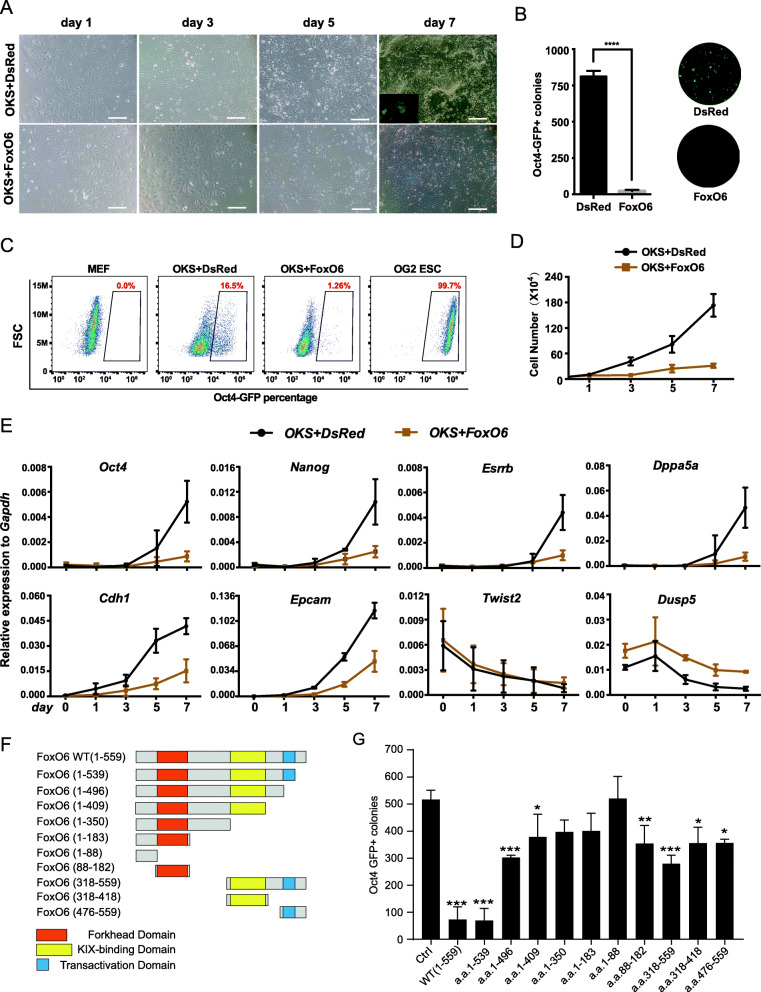
FoxO6 hinders somatic cell reprogramming. **a** Representative images showed the reprogramming process that MEFs were transduced with OKS + Ctrl and OKS + FoxO6. scale bars, 250 μm. **b** The number of Oct4-GFP colonies was counted at day 7 with or without FoxO6 in OKS mediated reprogramming system; n = 3. Representative fields of GFP+ colonies taken by fluorescence microscope in situ at day 7. Scale bar, 250 μm. **c** The proportions of Oct4-GFP positive colonies reflected reprogramming efficiency according to flow cytometry analysis. **d** Growth curves of OKS + Ctrl and OKS + FoxO6 induced conditions; n = 3. **e** RT-qPCR analysis showed the relative expression of representative genes related to pluripotency and MET process in reprogramming; n = 3. **f** Schematic diagrams illustrating the various truncated types of FoxO6. Forkhead domain, KIX-binding domain, and transactivation domain were shown as red rectangle, yellow rectangle and blue rectangle respectively. **g** Effects of the various truncated types of FoxO6 in OKS-mediated reprogramming; n = 3

**Fig. 4:**
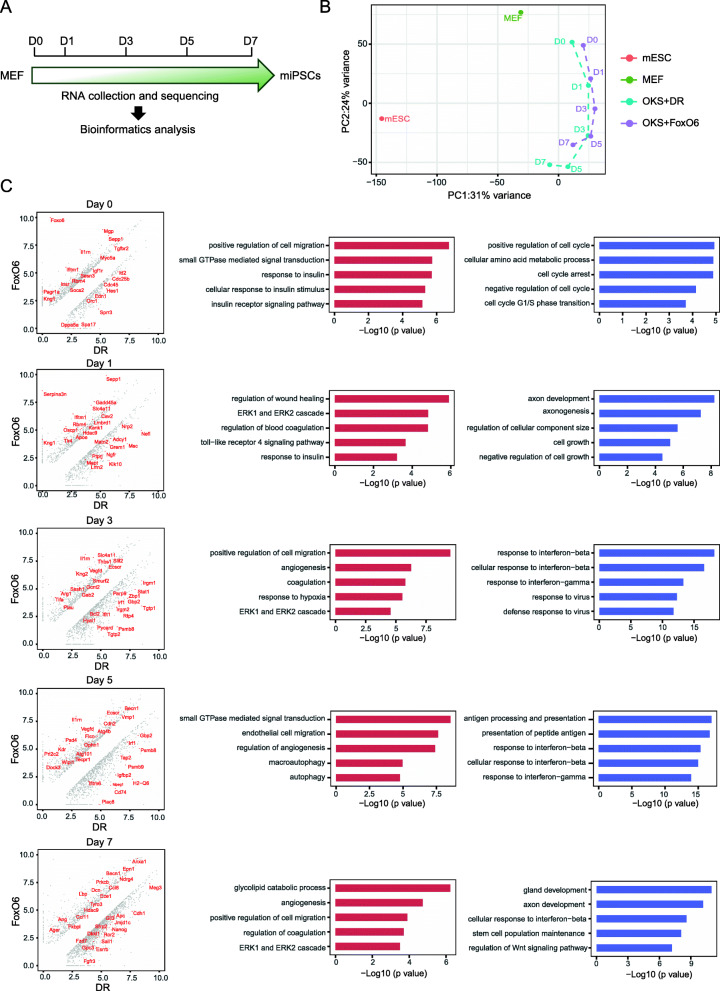
FoxO6 regulates mouse iPSCs induction. **a** Schematic of the bulk RNA-seq experimental design, we collected day 0/1/3/5/7 samples during the OKS + FoxO6 and OKS + DsRed reprogramming. **b** PCA analysis of the OKS + FoxO6 and OKS + DR mediated reprogramming, MEF and mESC. **c** Left: Scatter plot comparing the gene expression between he OKS + FoxO6 and OKS + DsRed populations at the day 0/1/3/5/7. The criteria for gene changes are FC > 2. Right: The -log10(*p* value) of the gene ontology (GO) term enriched in the left scatter plot. (Red is the genes highly expressed in OKS + FoxO6 than in OKS + DsRed, blue is opposite)

Among Fox transcription factors, FoxO6 hindered reprogramming most significantly. We are curious about how FoxO6 inhibits pluripotency induction. Based on cellular morphological changes daily observation during reprogramming, we hypothesized that FoxO6 might repress cell proliferation and mesenchymal to epithelial transition (MET) which is an essential process in early stage of somatic cell reprogramming. We collected cellular samples at day1/3/5/7 and calculated cell number which show that FoxO6 inhibited cell proliferation during OKS-mediated reprogramming compared with control group (Fig. 3a-d). Meanwhile, we performed realtime-qPCR for timecourse cellular samples and showed that FoxO6 suppressed upregulation of epithelial genes such as Cdh1, and Epcam and activation of pluripotent genes like Oct4, Nanog, Esrrb and Dppa5a, in accordance with cellular observation (Fig. 3e). As FoxO6 regulates gene expression through Forkhead DNA binding domain, KIX domain and transactivation domain, we wonder which domain plays crucial roles in reprogramming (Fig. 3f). To test this, we constructed a series of truncations of FoxO6 and examined them in reprogramming. The result revealed that each domain of FoxO6 could inhibit reprogramming variably and full length of FoxO6 suppress most obviously, suggesting that inhibitory effect of FoxO6 on reprogramming is dependent on all of three functional domains (Fig. 3g). To further investigate mechanisms of FoxO6 inhibiting somatic cell reprogramming, we firstly collected cell samples at day0/1/3/5/7 during reprogramming for RNA sequencing (RNA-seq) (Fig. 4a). Next, we compared the transcriptomes between OKS and OKS + FoxO6 mediated reprogramming systems by PCA analysis and found that the transcriptome of OKS + FoxO6 at day7 is close to that of OKS at day3, indicating that Foxo6 might delay reprogramming process (Fig. 4b). However, we extended OKS + FoxO6 reprogramming time but Oct4-GFP positive colonies didn’t increase (data not showed) which suggested FoxO6 didn’t delay but inhibit reprogramming through other mechanisms. Previous studies on cancer or other disease model demonstrated that FoxO6 affects cell fate transition through regulation of glucose metabolism, autophagy and cell cycle and is associated with muscle differentiation (Kim et al., 2013; van der Heide et al., 2005; van der Heide and Smidt, 2005). We made detailed analysis of timecourse RNA-seq data to get potential mechanisms of FoxO6 in reprogramming and novel function of FoxO6 in cell fate transition. Consistent with previous studies, FoxO6 promotes autophagy process and represses cell cycle gene expression (Supplementary Figure 3A-C). However, the whole reprogramming for OKS + FoxO6 is not associated with muscle differentiation or other germ layer fate commitment (Supplementary Figure 3D). Instead, FoxO6 tend to activate some epigenetic repressor for reprogramming like HDAC9, small GTPase mediated signal transduction and ERK pathway but suppress stem cell population maintenance, interferon-beta and Wnt pathway based on Gene ontology (GO) analysis (Fig. 4c). In addition, we analyzed specific genes of up-regulation or down-regulation for OKS and OKS + FoxO6 respectively and showed that OKS + FoxO6 shared little genes specific for OKS (Supplementary Figure 4A-B). Taken together, the results suggested that FoxO6 inhibit but not delay reprogramming mainly through repression of MET process, pluripotent genes activation and cell proliferation. Besides, novel function of FoxO6 on cell fate decision might be involved with epigenetic regulation, signaling pathway like ERK, WNT and small GTPase which need to be further proved by additional evidence.

## Discussion

In the last decade, the iPSC technology has generated novel insight into roles of transcription factors in cell fate decision. One such insight is the discovery that many transcription factors were identified to reprogram somatic cells into the lineage specific fate or pluripotent state and related mechanisms. Although Fox transcription factors were characterized to lineage commitment, transdifferentiation and adult stem cell maintenance, their functions on pluripotency induction and regulation is unknown. In this report, we show that the Fox family of transcription factors had a previously uncovered role in the induction of pluripotency based on OKS mediated iPSC technology. FoxD3, FoxD4 and FoxG1 not only enhance induced iPSCs generation from mouse fibroblasts but also substituted for Oct4 respectively and generate iPSCs with germline transmission. These results suggest that FoxD3, FoxD4 and FoxG1 could act as pluripotent inducers to construct a novel alternative reprogramming system like non-Yamanaka factors mediated reprogramming system previously reported by us and other labs. However, FoxA and FoxO subfamily genes repress this process obviously. As representative, FoxO6 inhibits cell proliferation, suppresses pluripotent genes activation and hinders the MET initiation during reprogramming. Whether they work as pioneer factors to alter the landscape of chromatin accessibility or other epigenetic regulators to enhance or suppress reprogramming is still unknown. Therefore, similar to diverse functions during development, Fox family of transcription factors play multiple roles in pluripotency induction and further studies are needed to dissect mechanisms undoubtedly to offer new insights for cell fate transition.

## Methods

### Mice

Mouse embryonic fibroblasts (MEFs) carrying the Oct4-GFP reporter were isolated from E13.5 embryos. MEFs and Plate-E cells were cultured in DMEM (Hyclone, high glucose) supplement with 10% FBS (Natocor), GlutaMAX (Gibco), and non-essential amino acids (Gibco). Mouse iPSCs and ESCs were maintained in serum-containing FBS medium (DMEM supplement with 10% FBS (Gibco), 1× GlutMAX, 1× NEAA, 1× Sodium pyruvate (Gibco), 0.1 mM β-mercaptoethanol (Gibco), PD03325901 (1 μM, In house-synthesized), CHIR99021 (3 μM, In house-synthesized), 1000 units/ml LIF (Millipore). All of the cell lines have been confirmed as mycoplasma contamination free with kit from Lonza (LT07–318).

### Generation with iPSCs

MEFs were reprogrammed according to previous reported protocols. MEFs with 3 passages were plated at 1 × 104 per well for OKS combination (24 well plate) or 3 × 104 per well for KS and FoxD3, and then infected with retrovirus generated from PlatE cells for two rounds. After two rounds infections, MEFs were induced with iCD1 medium. The number of GFP-Oct4 positive colonies was counted under microscope after several days inductions.

### FACS analysis

Cells were digested by 0.25% trypsin and resuspended with PBS + 1% FBS. Then, cells were analyzed the C6 flow cytometer (Becton Dickinson).

### Real-time PCR and RNA-seq

Cells were lysed with TRIzol reagent. ReverTra Ace (Toyobo) and oligo-dT (Takara) were used to synthesize cDNAs. Quantitative PCR was performed using Premix Ex TaqTM (Takara). q-PCR primer was displayed in Table 1. TruSeq RNA Sample Prep Kit (RS-122-2001, Illumina) was used for library construction and the Miseq Reagent Kit V2 (MS-102-2001, Illumina) was used for RNA-seq.

**Table 1:** Realtime-qPCR primer

Gene	Forward primer	Reverse primer
*Gapdh*	AACTTTGGCATTGTGGAAGGGCTCA	TTGGCAGCACCAGTGGATGCAGGGA
*Oct4*	CATTGAGAACCGTGTGAG	TGAGTGATCTGCTGTAGG
*Nanog*	CTCAAGTCCTGAGGCTGACA	TGAAACCTGTCCTTGAGTGC
*Dppa5a*	CCGTGCGTGGTGGATAAG	GCGACTGGACCTGGAATAC
*Sox2*	AGGGCTGGGAGAAAGAAGAG	CCGCGATTGTTGTGATTAGT
*Sall4*	CTAAGGAGGAAGAGGAGAG	CAAGGCTATGGTCACAAG
*Rex1*	CAGCCAGACCACCATCTGTC	GTCTCCGATTTGCATATCTCCTG
*Twist2*	AGATGACCAGCTGCAGCTAC	ATGTGCAGGTGGGTCCTG
*Esrrb*	TTTCTGGAACCCATGGAGAG	AGCCAGCACCTCCTTCTACA
*Cdh1*	CAGCCTTCTTTTCGGAAGACT	GGTAGACAGCTCCCTATGACTG
*Epcam*	CTTGTGTCTGCACGACCTGT	CCAAGCATTTAGACGCCAGTTT
*Dusp5*	GCCACCATCTGCCTTGCTTAC	ACTCCGCCTCTGCTTCACAA
*endo Sox2*	TGCGCCCAGTAGACTGCAC	ACCCCTCCCAATTCCCTTGTAT
*endo Oct4*	TCTTTCCACCAGGCCCCCGGCTC	TGCGGGCGGACATGGGGAGATCC

### Immunofluorescence

Cells were cultured on coverslips and fixed with 4% paraformaldehyde for 30 min. 0.1% Triton X-100 and 3% BSA were used to penetrate and block. Then, the cells were incubated with primary antibody for two hours in room temperature and washed with PBS for three times. Next, the cells were incubated with second antibody for one hour, washed with PBS for four times and then incubated with DAPI for 1 min. Finally, the coverslips were mounted on the slides for observation under fluorescence microscope. The primary antibodies diluted with 3% BSA were anti-Oct4 (SC-5279, 1: 200), anti-Nanog (BETHYL no. A300-397A, 1: 200), and anti-SSEA1 (RD, MAB2155, 1:100).

### Teratoma detection

KSFoxD3-iPSCs were digested with 0.05% trypsin and resuspended with DMEM/F-12 and Matrigel matrix. Approximately 1× 10^6^ cells were inoculated into the subcutaneous tissue of NOD-SCID mice. After two weeks injection, the teratoma was formed and fixed with 4% paraformaldehyde. Then, tumor samples were sectioned and stained.

### Generation of chimaeric mice

To generate chimaeras, mouse iPSCs were injected into ICR blastocysts and transplanted into pseudopregnant ICR females. The resulting chimaeric mice were determined for germline transmission by mating F2 mice with ICR mice.

### RNA-seq analysis

Sequenced reads were aligned to a transcriptome index generated from the GENCODE annotations transcriptome (M13), using RSEM (Li and Dewey, 2011), bowtie2 (Langmead and Salzberg, 2012). The TPM (Transcripts Per Kilobase Million) values was used for downstream analysis.

The R/Bioconductor v3.6.1 was used to detect the differentially expressed genes between the different samples. Fold change of > 2 were used as the threshold. Principal Component Analysis (PCA) was applied based on top 5000 of highly variable genes. Gene ontology (GO) analysis was performed using R package clusterProfiler (Yu et al.,^ 2012^). The associated plots were generated using the ggplot2 package(v3.3.2).

### Single cell RNA-seq analysis

Gene expression matrix was download from Sonja Nowotschin, 2019. Downstream analysis were performed with the Python package scanpy v1.4.5 (Wolf et al., 2018). The mouse early embryo(E3.5-E7.5) was selected with 54,952 cells. The dotplot was performed using sc.pl.dotplot as implemented in scanpy.

### Data availability

The RNA-seq data reported in this study was deposited with the gene expression omnibus with the accession number GEO: GSE161057.

The accession number for the 3F, 7F and CiPSC RNA-seq data described in this paper is GEO: GSE93029, GSE127927, GSE110264.

## Supplementary Information

**Additional file 1: Supplementary figure 1.** Fox family members expression during miPSCs induction and early development. (A) The expression of Fox family transcription factors in CiPSCs, 7F and OKS reprogramming by RNA-seq. CiPSCs from Cao et al., 7F from Wang et al. MEF, mESC and OKS from Guo et al. (B) The expression of Fox family transcription factors during mouse grastrulation. (C) Representative images of OKS + DsRed, OKS + FoxD3, OKS + FoxD4 and OKS + FoxG1 mediated reprogramming at day3 and day5. scale bars, 500 μm. (D) Growth curves of OKS + DsRed, OKS + FoxD3, OKS + FoxD4 and OKS + FoxG1 induced conditions; *n* = 3. (E) RT-qPCR analysis showed the relative expression of pluripotency core network and epithelial associated genes OKS + DsRed, OKS + FoxD3, OKS + FoxD4 and OKS + FoxG1 mediated reprogramming at day3 and day5; n = 3.

**Additional file 2: Supplementary figure 2.** (related to Fig. 1). The proportions of Oct4-GFP positive colonies reflected reprogramming efficiency at day 7 after induction according to flow cytometry analysis.

**Additional file 3: Supplementary figure 3.** (related to Fig. 4). Heatmaps for the expression of represented genes for cell cycle(A), pluripotency(B), mesenchymal-epithelial transition(C) and ectoderm-mesoderm-endoderm(D) during the OKS + FoxO6 and OKS + DR reprogramming process.

**Additional file 4: Supplementary figure 4.** (related to Fig. 4). (A) Heatmaps showing the expression levels of specifically upregulated/downregulated genes at the day 0/1/3/5/7 during OKS + FoxO6 and OKS + DR reprogramming. Right side of each heatmap are the relatived genes. (B) Venn diagram between genes in OKS + FoxO6 and OKS + DR cells. The genes up/down at each timepoint is showing in A.
