# Supplementary figures and images for: Forkhead box family transcription factors as versatile regulators for cellular reprogramming to pluripotency
Source: Cell Regen. 2021 Jul 2;10:17. doi: 10.1186/s13619-021-00078-4 (PMC8249537; doi:10.1186/s13619-021-00078-4)

Supplementary figure 1

A

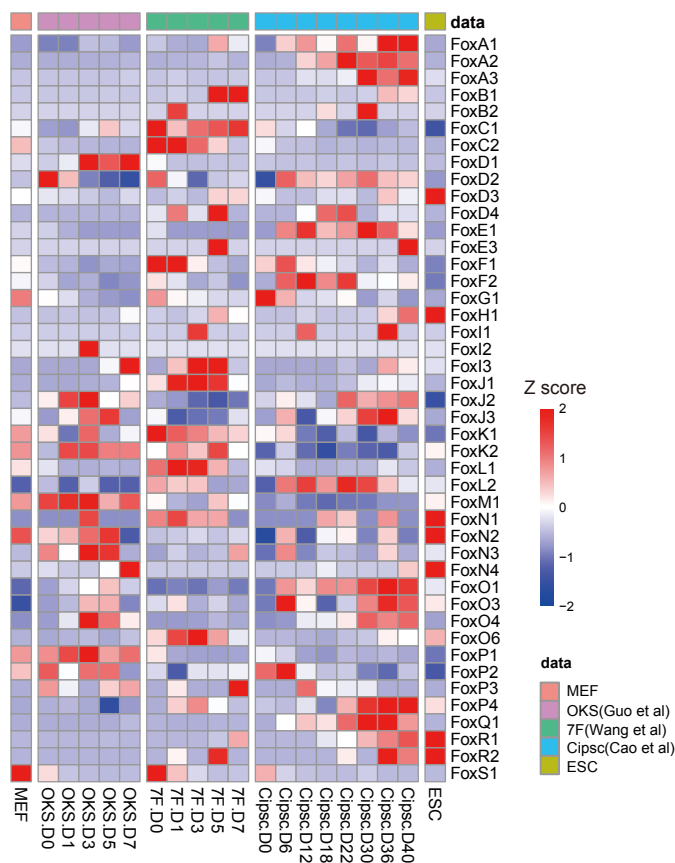

B

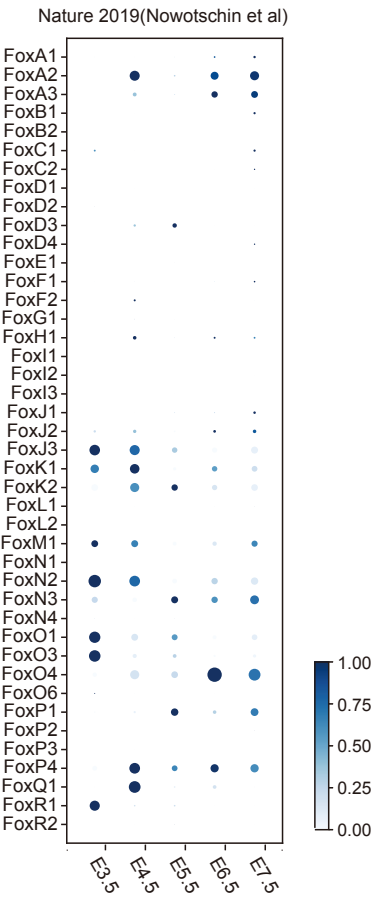

C

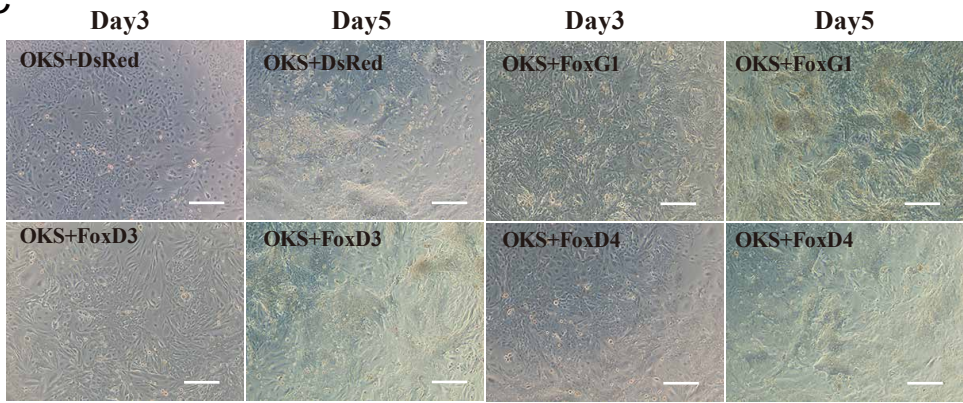

D

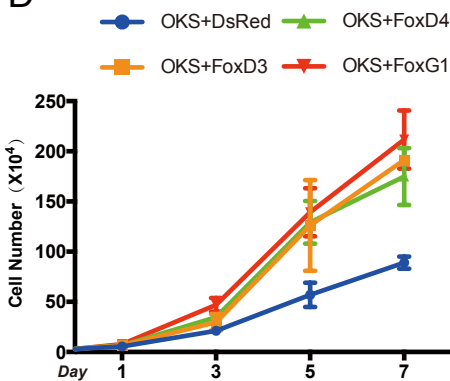

E

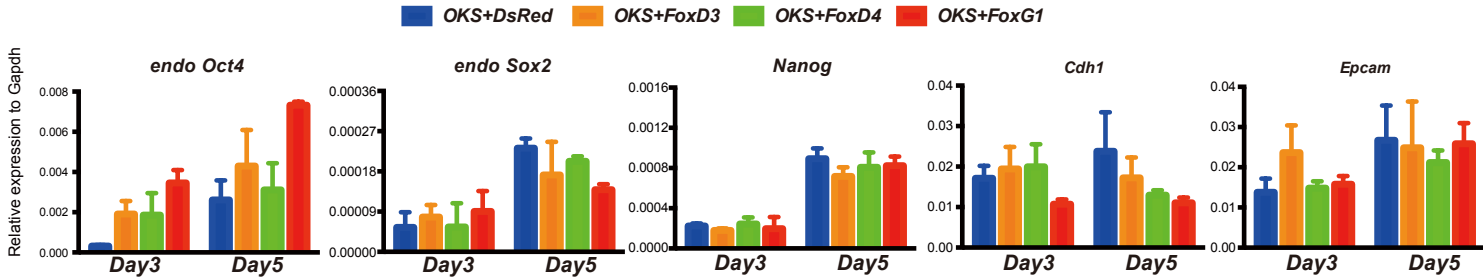

Supplement: Supplementary file 1 — Additional file 1: Supplementary figure 1. Fox family members expression during miPSCs induction and early development. (A) The expression of Fox family transcription factors in CiPSCs, 7F and OKS reprogramming by RNA-seq. CiPSCs from Cao et al., 7F from Wang et al. MEF, mESC and OKS from Guo et al. (B) The expression of Fox family transcription factors during mouse grastrulation. (C) Representative images of OKS + DsRed, OKS + FoxD3, OKS + FoxD4 and OKS + FoxG1 mediated reprogramming at day3 and day5. scale bars, 500 μm. (D) Growth curves of OKS + DsRed, OKS + FoxD3, OKS + FoxD4 and OKS + FoxG1 induced conditions; n = 3. (E) RT-qPCR analysis showed the relative expression of pluripotency core network and epithelial associated genes OKS + DsRed, OKS + FoxD3, OKS + FoxD4 and OKS + FoxG1 mediated reprogramming at day3 and day5; n = 3. [file 13619_2021_78_MOESM1_ESM.pdf]

### Supplementary figure 2

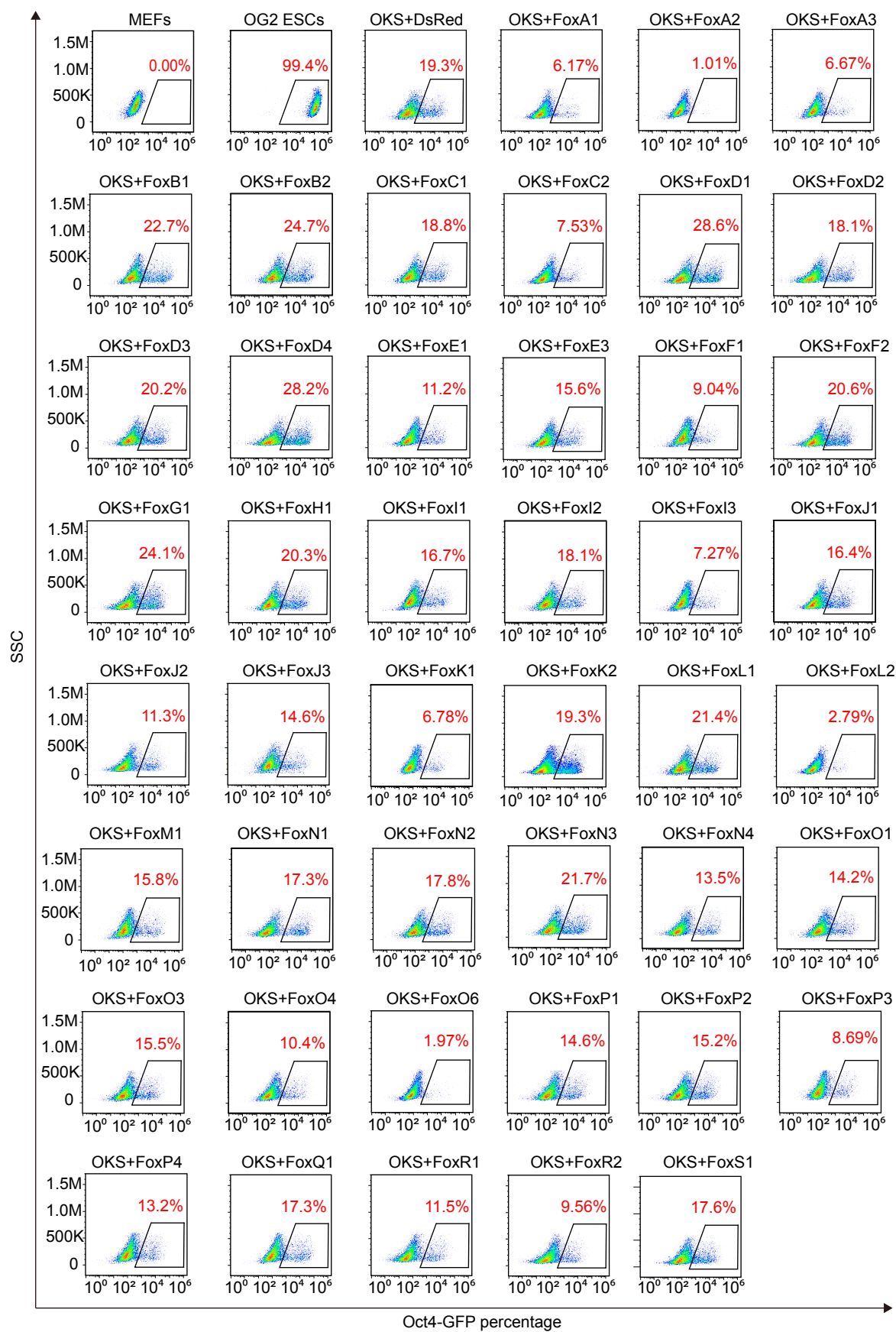

Supplement: Supplementary file 2 — Additional file 2: Supplementary figure 2. (related to Fig. 1). The proportions of Oct4-GFP positive colonies reflected reprogramming efficiency at day 7 after induction according to flow cytometry analysis. [file 13619_2021_78_MOESM2_ESM.pdf]

Supplementary figure 3

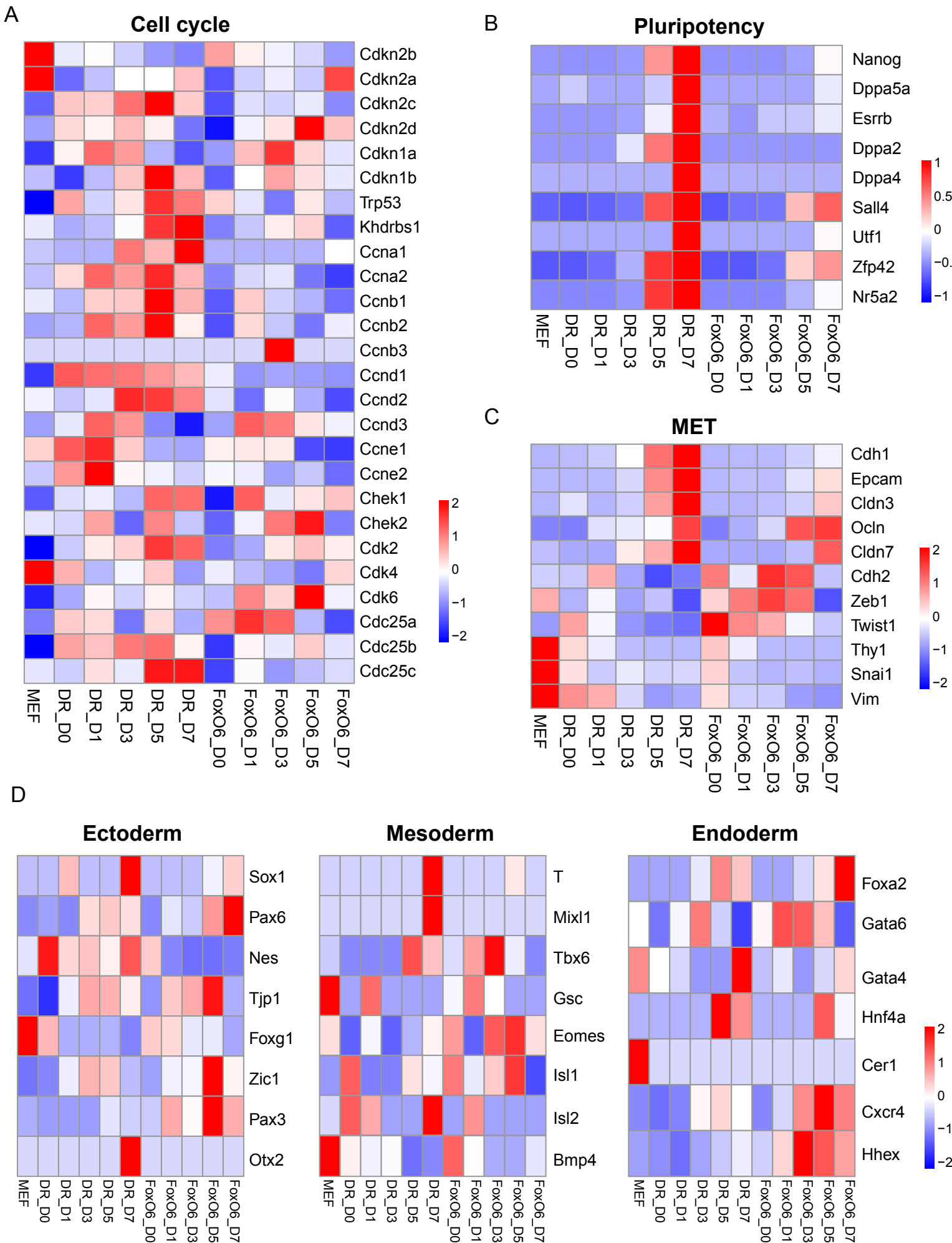

Supplement: Supplementary file 3 — Additional file 3: Supplementary figure 3. (related to Fig. 4). Heatmaps for the expression of represented genes for cell cycle(A), pluripotency(B), mesenchymal-epithelial transition(C) and ectoderm-mesoderm-endoderm(D) during the OKS + FoxO6 and OKS + DR reprogramming process. [file 13619_2021_78_MOESM3_ESM.pdf]

Supplementary figure 4

A

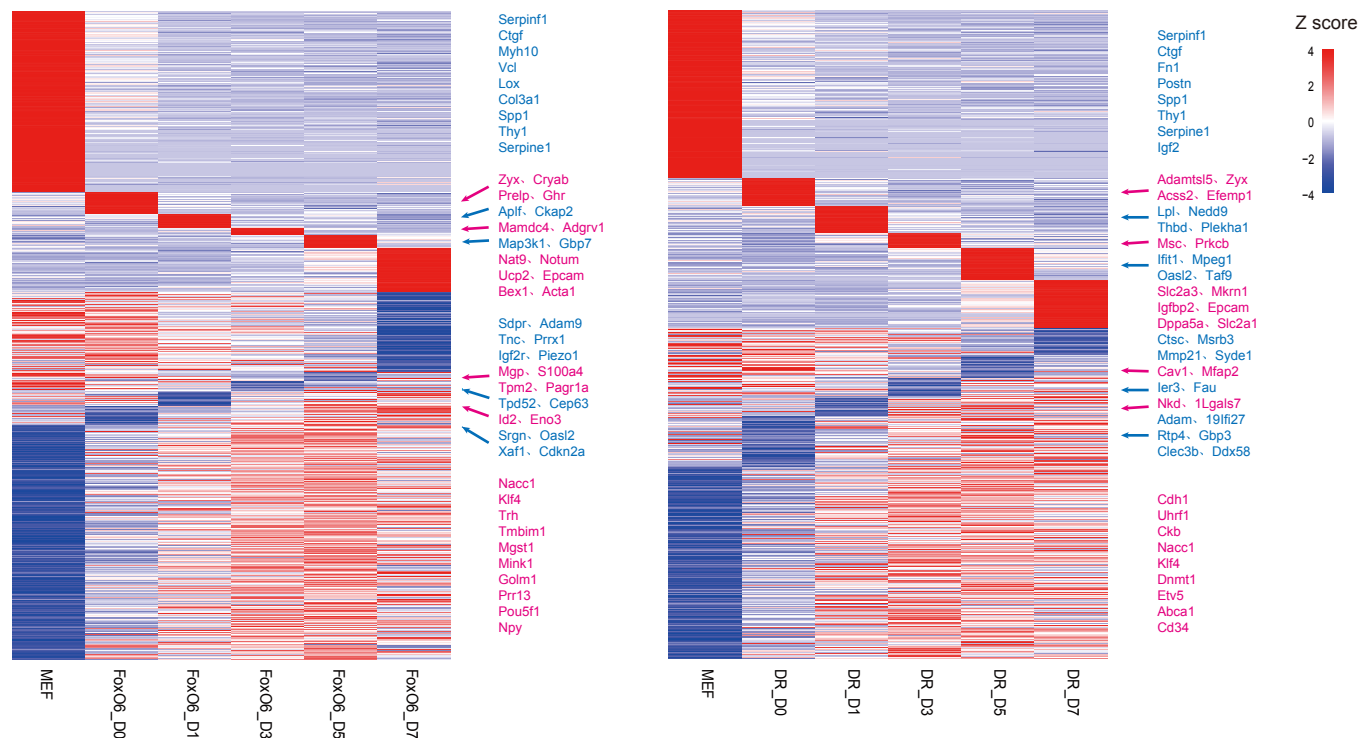

B

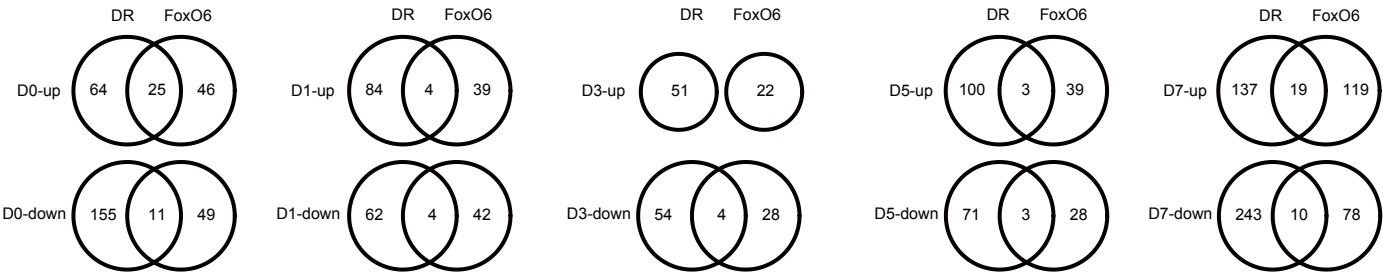

Supplement: Supplementary file 4 — Additional file 4: Supplementary figure 4. (related to Fig. 4). (A) Heatmaps showing the expression levels of specifically upregulated/downregulated genes at the day 0/1/3/5/7 during OKS + FoxO6 and OKS + DR reprogramming. Right side of each heatmap are the relatived genes. (B) Venn diagram between genes in OKS + FoxO6 and OKS + DR cells. The genes up/down at each timepoint is showing in A. [file 13619_2021_78_MOESM4_ESM.pdf]
